# Three Peptide Modulators of the Human Voltage-Gated Sodium Channel 1.7, an Important Analgesic Target, from the Venom of an Australian Tarantula

**DOI:** 10.3390/toxins7072494

**Published:** 2015-06-30

**Authors:** Chun Yuen Chow, Ben Cristofori-Armstrong, Eivind A. B. Undheim, Glenn F. King, Lachlan D. Rash

**Affiliations:** The University of Queensland, Institute for Molecular Bioscience, St Lucia, Queensland 4072, Australia; E-Mails: chun.chow@uqconnect.edu.au (C.Y.C.); b.cristoforiarmstrong@uq.edu.au (B.C.-A.); e.undheim@imb.uq.edu.au (E.A.B.U.)

**Keywords:** *Phlogius* sp., spider venom, venom peptide, voltage-gated sodium channel, Na_V_1.7, two-electrode voltage clamp electrophysiology, ion channel, mass spectrometry

## Abstract

Voltage-gated sodium (Na_V_) channels are responsible for propagating action potentials in excitable cells. Na_V_1.7 plays a crucial role in the human pain signalling pathway and it is an important therapeutic target for treatment of chronic pain. Numerous spider venom peptides have been shown to modulate the activity of Na_V_ channels and these peptides represent a rich source of research tools and therapeutic lead molecules. The aim of this study was to determine the diversity of Na_V_1.7-active peptides in the venom of an Australian *Phlogius* sp. tarantula and to characterise their potency and subtype selectivity. We isolated three novel peptides, μ-TRTX-Phlo1a, -Phlo1b and -Phlo2a, that inhibit human Na_V_1.7 (hNa_V_1.7). Phlo1a and Phlo1b are 35-residue peptides that differ by one amino acid and belong in NaSpTx family 2. The partial sequence of Phlo2a revealed extensive similarity with ProTx-II from NaSpTx family 3. Phlo1a and Phlo1b inhibit hNa_V_1.7 with IC_50_ values of 459 and 360 nM, respectively, with only minor inhibitory activity on rat Na_V_1.2 and hNa_V_1.5. Although similarly potent at hNa_V_1.7 (IC_50_ 333 nM), Phlo2a was less selective, as it also potently inhibited rNa_V_1.2 and hNa_V_1.5. All three peptides cause a depolarising shift in the voltage-dependence of hNa_V_1.7 activation.

## 1. Introduction

Na_V_ channels are responsible for propagating action potentials in excitable cells, most notably nerves and muscle [1]. As such they are important therapeutic targets for a wide variety of pathophysiological conditions, including chronic pain, cardiac arrhythmia, and epilepsy [2,3,4]. Humans and rodents contain a complex repertoire of nine Na_V_ channel subtypes denoted Na_V_1.1–Na_V_1.9. Several studies on the genetic basis underlying several striking human phenotypes have revealed the importance of human Na_V_1.7 (hNa_V_1.7) as an analgesic target. Gain-of-function mutations in the *SNC9A* gene that encodes hNa_V_1.7 lead to painful inherited neuropathies [5,6,7,8], whereas loss-of-function mutations result in a congenital indifference to all forms of pain [9]. Importantly, therapeutics targeted against Na_V_1.7 need to have high selectivity over other Na_V_ channel subtypes such as Na_V_1.5, which is critical for the cardiac action potential, and Na_V_1.6, which is essential for action potential generation at nodes of Ranvier in myelinated motor neurons [10,11].

Na_V_ channel pharmacology has been largely defined by neurotoxins from natural sources, including many venom-derived peptides [12,13]. The identification and characterisation of spider-venom peptides that selectively modulate the activity of Na_V_ channels (so-called NaSpTx peptides) has expanded our understanding of their mechanisms of action and provided templates for drug development. To date, twelve families of NaSpTx have been described based on the level of sequence conservation and disulfide-bond connectivity [14]. Some of these peptides demonstrate excellent affinity and specificity for particular Na_V_ channel isoforms [15], although none appears to be sufficiently selective for therapeutic use.

The majority of tarantula-venom peptides are 3.0–4.5 kDa in size and highly disulfide-bridged [16,17]. They typically adopt a highly stable inhibitor cystine knot (ICK) fold that provides resistance to chemical and thermal degradation as well as proteases, making them promising lead molecules for the development of ion channel therapeutics [18,19]. Although the increasing use of venom-gland transcriptomes has led to a rapid increase in the number of available venom-peptide sequences [20], the venoms of Australian tarantulas remain relatively unstudied. In the present study we report the amino acid sequence, potency and selectivity of three hNa_V_1.7-active peptides isolated from the venom of an unstudied Australian tarantula.

## 2. Results and Discussion

### 2.1. Assay-Guided Fractionation and Peptide Purification

**Figure 1:**
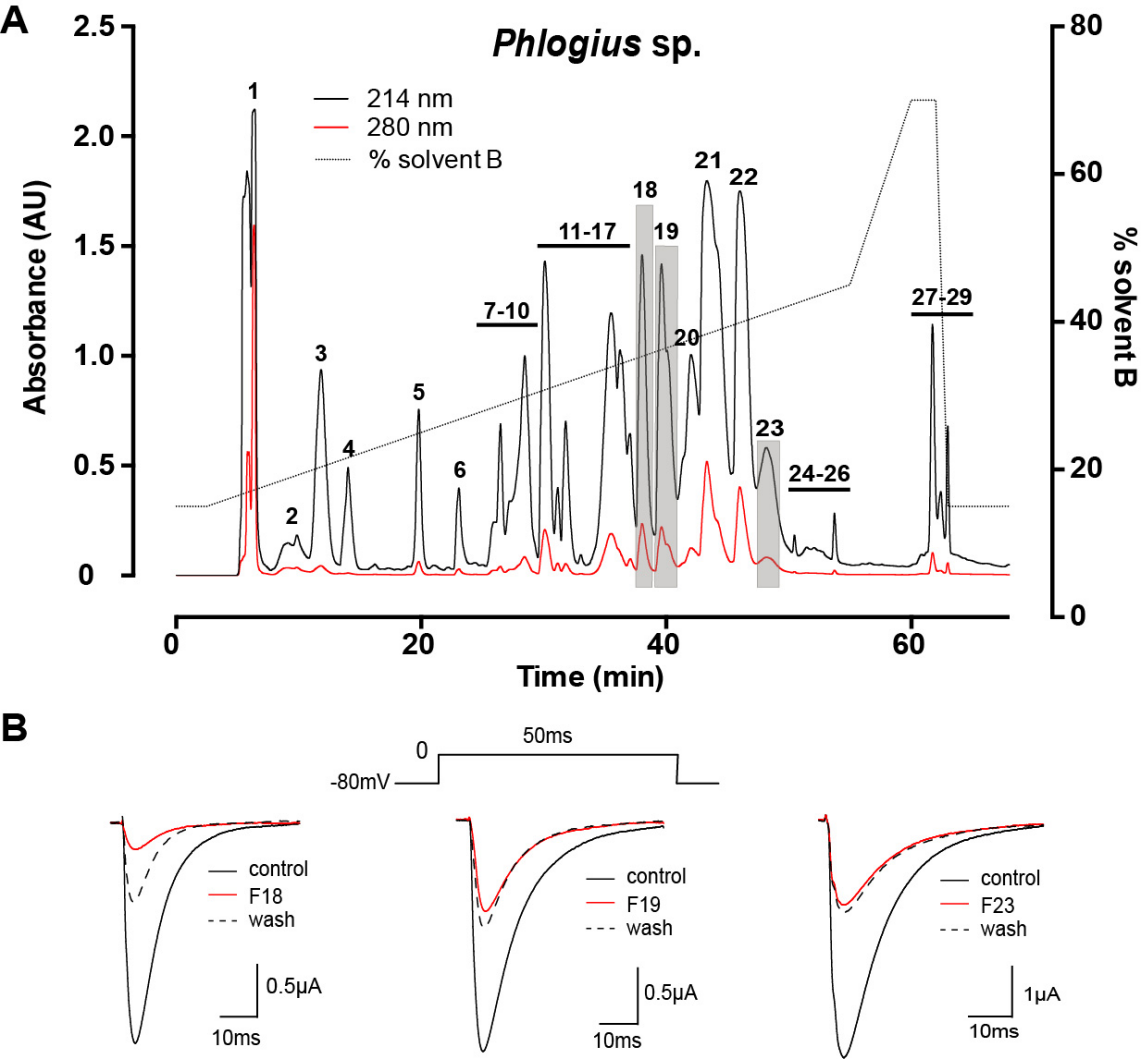
(**A**) Chromatogram resulting from fractionation of crude *Phlogius* sp. venom using C_18_ RP-HPLC. The numbers correspond to collected fractions, and active fractions are shaded grey; (**B**) Representative whole-cell current traces obtained from hNa_V_1.7 channels expressed in *Xenopus* oocytes. Current traces are shown in the absence and presence of F18, 19 and 23, and after ~3 min of peptide washout. Sodium currents were evoked using the voltage protocol shown above the central trace.

**Figure 2:**
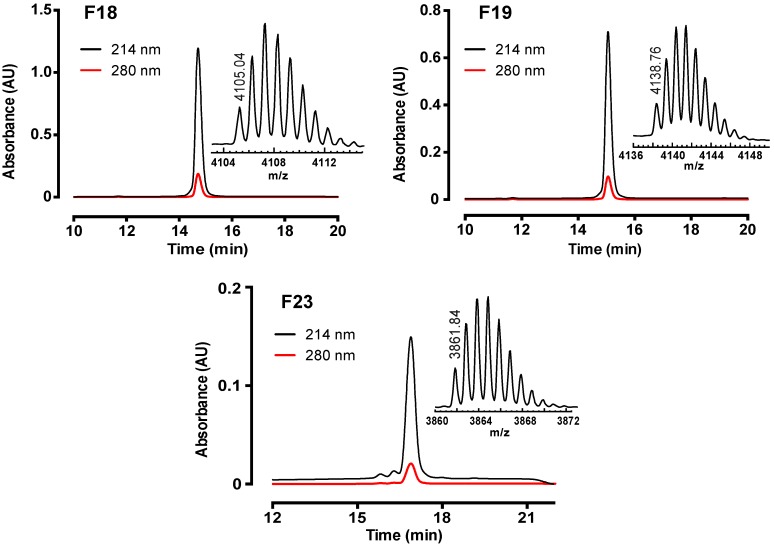
Chromatograms resulting from final purification of hNa_V_1.7-active peptides using C_18_ RP-HPLC. Absorbance was monitored at 214 and 280 nm. Inserts show MALDI-TOF mass spectra, with the monoisotopic M + H^+^ for each peptide indicated.

Female *Phlogius* sp. tarantulas from the Cairns region of northern Queensland, Australia were purchased from a commercial collector. Venom was acquired by electrostimulation of the chelicerae. Fractionation of crude venom using reversed-phase (RP) HPLC yielded 29 major fractions, indicating that *Phlogius* sp. venom is moderately complex (Figure 1A). The majority of components eluted between 25% and 40% solvent B (0.043% trifluoroacetic acid in 90% acetonitrile). Electrophysiological screening of each fraction against hNa_V_1.7 heterologously expressed in *Xenopus* oocytes resulted in the identification of three fractions (18, 19 and 23; highlighted in grey in Figure 1A) that inhibited hNa_V_1.7. Three pure peptides were isolated from these fractions using two subsequent steps of RP-HPLC fractionation on a C_18_ column. The final step of RP-HPLC fractionation resulted in a single peak for each active peptide (≥ 95% purity), and a single molecular ion by matrix-assisted laser desorption ionisation time-of-flight mass spectrometry (MALDI-TOF MS), which did not reveal other contaminants (Figure 2).

### 2.2. Peptide Sequence Determination

#### 2.2.1. Venom-Gland Transcriptome

A venom-gland transcriptome was obtained using venom-gland mRNA isolated from a single *Phlogius* sp. specimen. The transcriptomic data was used solely as a raw database to search for sequence matches to the proteomic data and it was not annotated.

#### 2.2.2. MALDI-TOF MS Using 1,5-DAN Matrix

**Figure 3:**
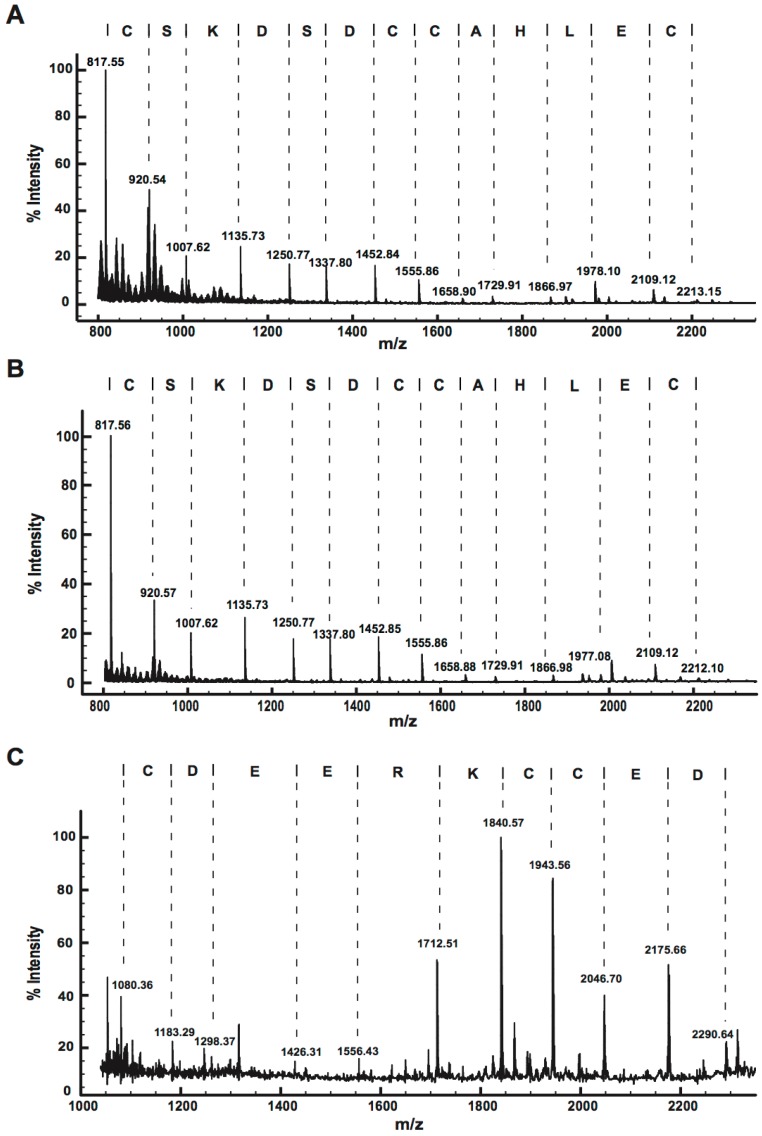
Positive-ion MALDI-ISD spectra of (**A**) F18, (**B**) F19, and (**C**) F23, obtained using 1,5-DAN matrix. The deduced peptide sequences are shown above the spectra.

The hydrogen-donating ability of 1,5-diaminonapthalene (1,5-DAN) causes partial reduction of cystines and enhances in-source decay (ISD) fragmentation in the laser plume, providing information on the number of disulfide bridges and fragments for *de novo* sequencing [21]. The MALDI-ISD spectra of peptides from F18, F19 and F23 (Figure 3) revealed a dominant series of *c* ions. The mass difference between the fragment ions was used to obtain sequence information for each peptide. An identical 13-residue sequence tag was obtained for F18 and F19, while a distinctly different 10-residue sequence tag was obtained for F23.

**Figure 4:**
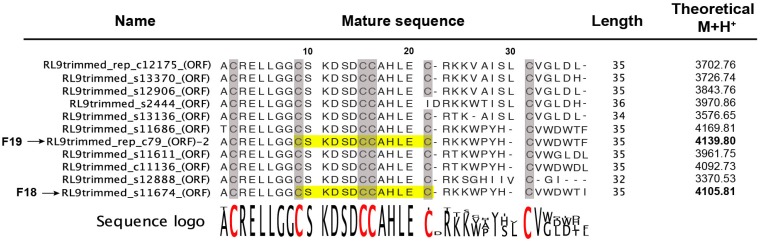
Alignment of mature toxin sequences obtained by BLAST search of the partial sequences of F18 and F19 obtained from MALDI-TOF MS (highlighted in yellow) against a *Phlogius* sp. venom-gland transcriptome. A sequence logo for this alignment is shown, with conserved Cys residues that form the ICK motif highlighted in red or shaded grey. The theoretical M + H^+^ mass is shown for each oxidised peptide (assuming non-amidated *C*-termini). The M + H^+^ values in bold are those for the sequences of F18 and F19.

A BLAST search was used to compare the sequence tag “CSKDSDCCAHLEC” obtained from MALDI-ISD spectra of F18 and F19 against the *Phlogius* venom-gland transcriptome. This resulted in matches with 11 mature peptide sequences with lengths varying from 32 to 36 residues (Figure 4), with the *C*-terminal region being less conserved than the *N*-terminal region. These predicted mature peptides each consist of six cysteine residues, and the intercysteine spacing is consistent with an ICK motif (*i.e.*, C–C–CC–C–C) [22]. The observed M + H^+^ of F18 (4105.04) was 0.77 mass units lower than the calculated M + H^+^ (4105.81) of one of the translated cDNA sequences, *RL9trimmed_s11674* (see Supplementary Figure S1 for cDNA and predicted prepropeptide sequences for F18 and F19). This suggests that F18 corresponds to this sequence but contains an amidated *C*-terminus, a common modification in spider-venom peptides that reduces the peptide mass by 1.0 Da. Similarly, the observed M + H^+^ of F19 (4138.76) was 1.04 mass units lower than one of the transcriptome-derived sequences (*RL9trimmed_rep_c79*) which only differs from the sequence identified for F18 by one residue at the *C*-terminus. This indicates that the F19 peptide is a paralog of F18 that contains an amidated phenylalanine at the *C*-terminus rather than an amidated isoleucine. This difference is consistent with the mass difference of +33.7 between F18 and F19 and the slightly longer RP-HPLC retention time for the F19 peptide. This sequence information was sufficient to name the peptides from F18 and F19 as μ-TRTX-Phlo1a (hereafter Phlo1a) and μ-TRTX-Phlo1b (hereafter Phlo1b), respectively, based on the rational nomenclature proposed for spider-venom peptides [20]. These 35-residue peptides share a high level of sequence similarity with tarantula-venom peptides in NaSpTx family 2, as discussed below.

Surprisingly, the RP-HPLC peak corresponding to F23 in the venom sample used for peptide isolation (Figure 1A) was absent in venom from the *Phlogius* specimen used to obtain the venom-gland transcriptome, even though the chromatograms of the two venoms were otherwise identical (data not shown). Thus, no additional sequence information was acquired from a BLAST search of the MS-derived F23 sequence against the venom-gland transcriptome. However, the MS-derived sequence tag obtained for F23 is similar to other mature toxins belonging to NaSpTx Family 3, as discussed below. Since F23 clearly belongs to a different toxin family than F18 and F19, it was named μ-TRTX-Phlo2a.

#### 2.2.3. MALDI-TOF MS Analysis of Tryptic Peptides

The three *Phlogius* peptides were reduced and alkylated using the volatile reagents triethyl-phosphine and iodoethanol, respectively, prior to tryptic digestion. The peptides eluted at a later RP-HPLC retention time following reduction/alkylation, presumably due to exposure of more hydrophobic side chains (data not shown). The mass of each peptide was found to increase by 270 Da following reduction/alkylation, consistent with the presence of six cysteine residues (*i.e.*, the addition of six ethanolyl groups of 45 Da each) that form three disulfide bonds.

**Figure 5:**
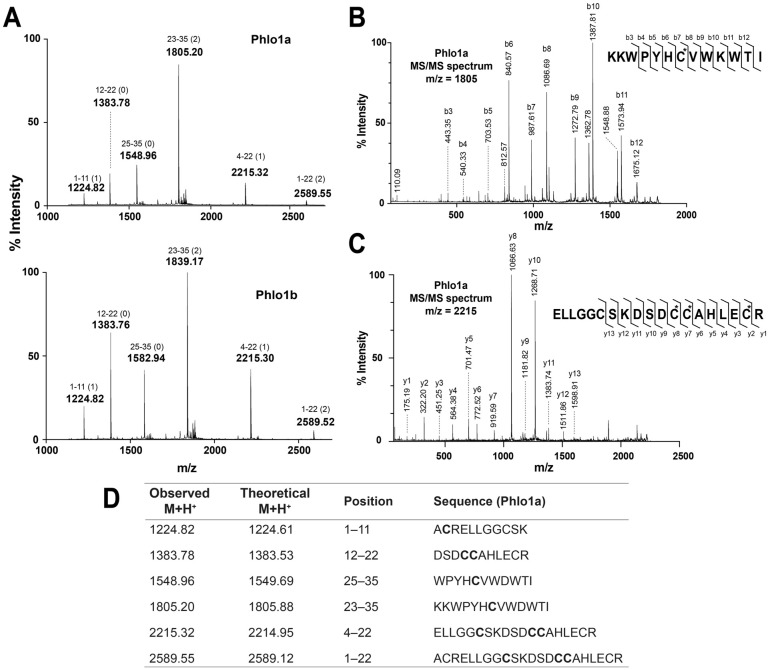
(**A**) MALDI-TOF mass spectra of tryptic digests of Phlo1a (upper panel) and Phlo1b (bottom panel). Amino acid positions (and number of missed cleavages) are indicated above the peak masses; (**B**) MS/MS analysis of the Phlo1a precursor ions 1805.20 and (**C**) 2215.32; **(D)** Comparison of the observed and theoretical M + H^+^ for the ions observed, their corresponding residue positions and fragment sequence.

To confirm the predicted sequences of Phlo1a and Phlo1b, the reduced/alkylated peptides were subjected to trypsin digestion and MS/MS sequencing. Peptide mass fingerprints (PMFs) of Phlo1a and Phlo1b show that four of the six observed digestion fragments for each peptide were identical (1224.82, 1383.77, 2215.32 and 2589.55) (Figure 5A) while the remaining two were 33.9 mass units higher for Phlo1b than Phlo1a, consistent with an Ile to Phe substitution. All ions observed from the tryptic digest match the theoretical digest values from the sequences obtained from the venom-gland transcriptome (Fig 5A,D) with the exceptions of 1548 and 1805 (for Phlo1a and the corresponding ions from Phlo1b), which are ~0.8 units less than the predicted masses (as determined with a free acid *C*-terminus), providing further evidence that the peptides are *C*-terminally amidated. The sequences of the fragments corresponding residues 4–22 and 23–35 were determined by MS/MS (Figure 5B,C) and they match the predicted sequences. Taken together, these results support the sequences of Phlo1a and Phlo1b predicted from the transcriptomic data (Figure 4).

**Figure 6:**
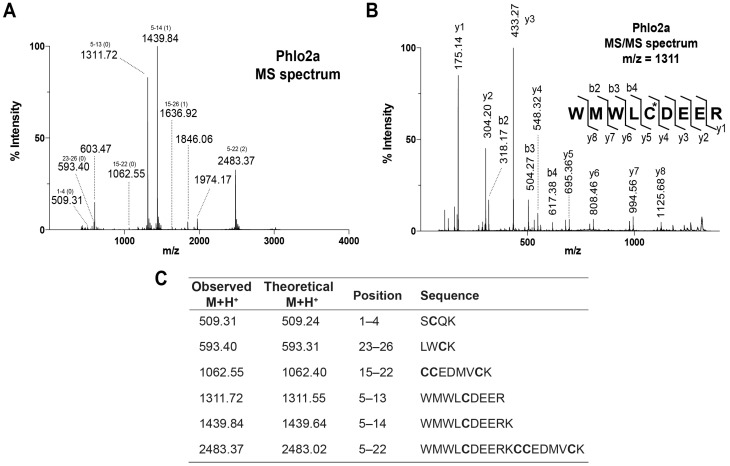
(**A**) MALDI-TOF MS analysis of peptides fragments from tryptic digest of Phlo2a. Amino acid positions and the number of missed cleavage are indicated above the peak masses. (**B**) MS/MS analysis of tryptic peptides with m/z 1311.72. (**C**) Comparison of observed and theoretical M + H^+^ for tryptic fragments of alkylated Phlo2a obtained using MALDI-TOF MS.

Phlo2a belongs to NaSpTx3, which is comprised entirely of short (29–33 residue) tarantula ICK peptides [14,16]. NaSpTx3 is characterised by 26 highly conserved *N*-terminal residues [YCQKWMWTCDxxRKCCE(G/D)(L/M)VCRLWC(K/R)] and a more variable *C*-terminal region often containing one or more of Lys, Arg, Ile or Leu. Based on this high level of sequence identity and the sequence tag obtained from 1,5 DAN MS showing that positions 11 and 12 are Glu, and position 18 is Asp, we predicted that Phlo2a has an *N*-terminal sequence of YCQKWMWTCDEERKCCED(L/M)VCRLWC(K/R) and compared our experimental data to this prediction. Tryptic digestion and MS analysis of Phlo2a (with ethanoylated Cys residues) revealed a fragment fingerprint that was somewhat consistent with this prediction (Figure 6A). MS/MS analysis of several fragment ions revealed that positions 8, 19 and 26 are Leu, Met and Lys, respectively (Figure 6B,C). The main exception to our prediction was the *N*-terminal four residues, which with a Tyr was predicted to have an m/z of 570.22; however, this ion was not present. *N*-terminal sequencing of another NaSpTx3 family member by Edman degradation showed that the *N*-terminal Tyr can be substituted by a Ser (L.D. Rash, unpublished observation). Using an *N*-terminal Ser residue and the corrected residues at 8, 19 and 26, we obtain complete agreement between the ions observed in the 1,5-DAN mass spectra, the trypsin digest, and MS/MS fragments and the theoretical values for these ions for the first 26 residues of Phlo2a (Figure 6C).

#### 2.2.4. Ladder Sequencing Using Carboxypeptidase Y

**Figure 7:**
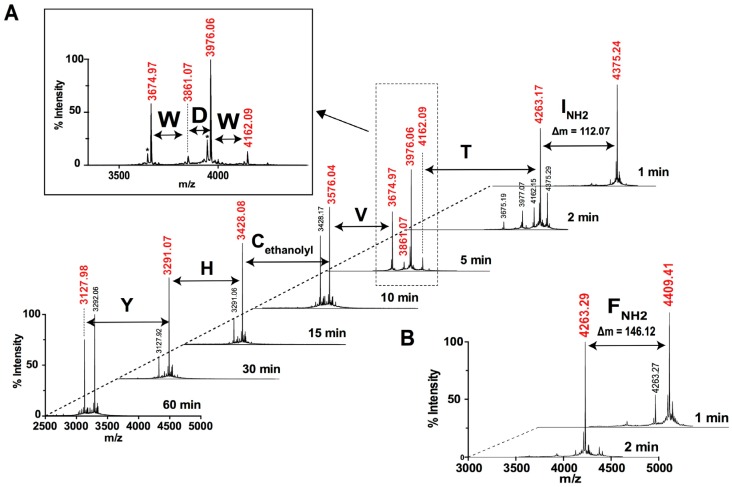
MALDI-TOF mass spectra obtained at different times points (from 1 to 60 min) during CPY digestion of reduced/alkylated (**A**) Phlo1a and (**B**) Phlo1b.

MS analysis of peptides resulting from tryptic digest of Phlo1a and Phlo1b confirmed almost the entire mature toxin sequence predicted from the venom-gland transcriptome, and suggest that the *C*-terminal residue is amidated. In order to confirm the nature of the *C*-termini, the reduced/alkylated peptides were digested with carboxypeptidase Y (CPY), an exopeptidase that cleaves one residue at a time from the *C*-terminus. MALDI TOF MS analysis of the CPY digestion of Phlo1a and Phlo1b taken over a period of 60 min provided experimental evidence for the sequence of the nine last amino acid residues and confirmed that the *C*-terminal residues are indeed amidated (Figure 7). In the case of Phlo1a, the observed *C*-terminal residue mass was 112.07, exactly 1 unit less than the theoretical residue mass of isoleucine or the isobaric leucine with a carboxylic acid. However, a search against the venom-gland transcriptome only revealed a match with a *C*-terminal isoleucine, and hence we concluded that this must be the *C*-terminal residue in Phlo1a. Likewise, CPY digestion clearly revealed that the *C*-terminal residue of Phlo1b is phenylalanine-amide (146.12 as opposed to the free acid residue mass of 147.07).

**Figure 8:**
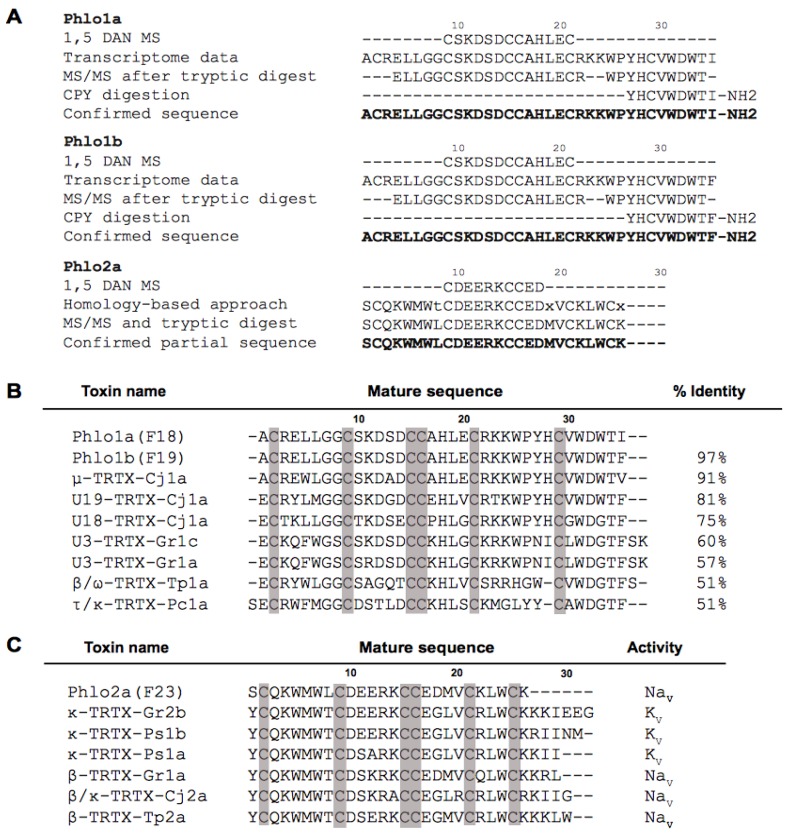
(**A**) Summary of the experimental evidence for amino acid sequences of Phlo1a and Phlo1b, and partial sequence of Phlo2a, in comparison to predictions from the venom-gland transcriptome (confirmed sequence in bold); (**B**) Sequence alignment of Phlo1a and Phlo1b with other members of the NaSpTx2; (**C**) Sequence alignment of Phlo2a with other members of the NaSpTx3. Cysteine residues are shaded.

Approximately 12% of spider toxins are *C*-terminally amidated [23]. In addition to a possible role in peptide stability, *C*-terminal amidation can modulate biological activity. The 35-residue spider-venom peptide huwentoxin-IV (HwTx-IV) is a member of NaSpTx Family 1 isolated from venom of the tarantula *Haplopelma schmidti* (formerly known as *Ornithoctonus huwena*) [24]. Remarkably, native HwTx-IV with *C*-terminal amidation inhibits hNa_V_1.7 with is ~50-fold higher potency than a recombinant version with a *C*-terminal carboxylate group. Although not in the same peptide family as HwTx-IV (NaSpTx Family 1), amidation might have substantial effects on the potency and selectivity of Phlo1a and Phlo1b and this should be examined in future studies. The experimental evidence for the complete sequences of Phlo1a and Phlo1b and the *N*-terminal sequence of Phlo2a is summarised in Figure 8. The verified sequences confirm our classification of Phlo1a and Phlo1b into NaSpTx2. Family 2 peptides range in length from 33 to 41 residues with three disulfide bonds and they constitute the largest family of spider toxins that inhibit Na_V_ channels (Figure 8B) [14]. The most similar toxin to Phlo1a/1b with 91% identity is μ-theraphotoxin-Cj1a (91%), a Na_V_ channel modulator from venom of the tarantula *Chilobrachys guangxiensis* [25]. Additionally, Phlo1a shares 51% sequence identity with β/ω-TRTX-Tp1a (ProTx-1) from venom of the tarantula *Thrixopelma pruriens*, a potent blocker of human Na_V_1.5, Na_V_1.7 and Na_V_1.8 channels [26].

### 2.3. Electrophysiological Characterisation of Phlogius Peptides

#### 2.3.1. Effects of Phlo1a, Phlo1b and Phlo2a on hNa_V_1.7 Currents

**Figure 9:**
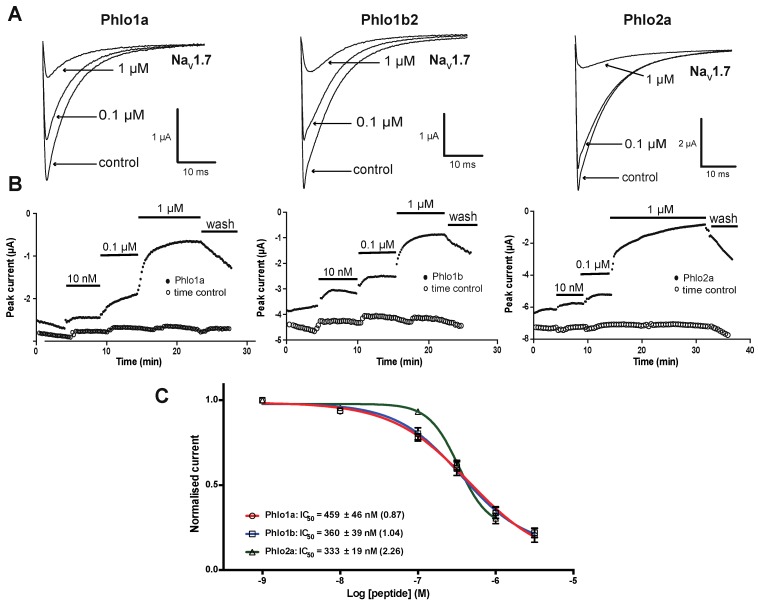
Effects of *Phlogius* peptides on hNa_V_1.7 expressed in oocytes. (**A**) Whole-cell current traces in absence (control) and presence of 0.1 or 1 μM peptide. Currents were evoked by a 50-ms step depolarisation to 0 mV from a holding potential of −80 mV every 10 s. (**B**) Time course for inhibition of hNa_V_1.7 by different peptide concentrations. Time controls show stable current amplitude in the absence of peptide. (**C**) Concentration-effect curves for inhibition of hNa_V_1.7 by Phlo1a, Phlo1b and Phlo2a (*n* = 5–7). Data are mean ± S.E.M. Hill coefficients are shown in parentheses.

We investigated the ability of Phlo1a, Phlo1b and Phlo2a to inhibit currents carried by hNa_V_1.7 channels heterologously expressed in *Xenopus* oocytes using two-electrode voltage-clamp (TEVC) electrophysiology. The three peptides inhibited hNa_V_1.7 in a concentration-dependent manner (Figure 9). Phlo1a and Phlo1b, which are identical except for their *C*-terminal residue, inhibited hNa_V_1.7 with similar potency (IC_50_ values of 459 and 360 nM, respectively) (Figure 9C), indicating that the *C*-terminal residue is not critical for interaction with hNa_V_1.7. Phlo2a, which belongs to NaSpTx Family 3, inhibited hNa_V_1.7 with an IC_50_ of 333 nM, making all three peptides similarly potent on hNa_V_1.7 (Figure 9). After application of 1 μM Phlo2a, the current level had not plateaued after 20 min. Notably, the concentration-effect curve for Phlo2a inhibition of hNa_V_1.7 currents was steeper compared with that of Phlo1a and Phlo1b, suggesting that it may bind to the channel at multiple sites with positive cooperativity. Several spider toxins have been shown to bind multiple sites on vertebrate Na_V_ channels; for example, ProTx-II binds to the voltage sensors in domains I, II and IV of rat Na_V_1.2 [27].

#### 2.3.2. Effect of Phlo1a, Phlo1b and Phlo2a on the Current-Voltage Relationship for hNa_V_1.7

**Figure 10:**
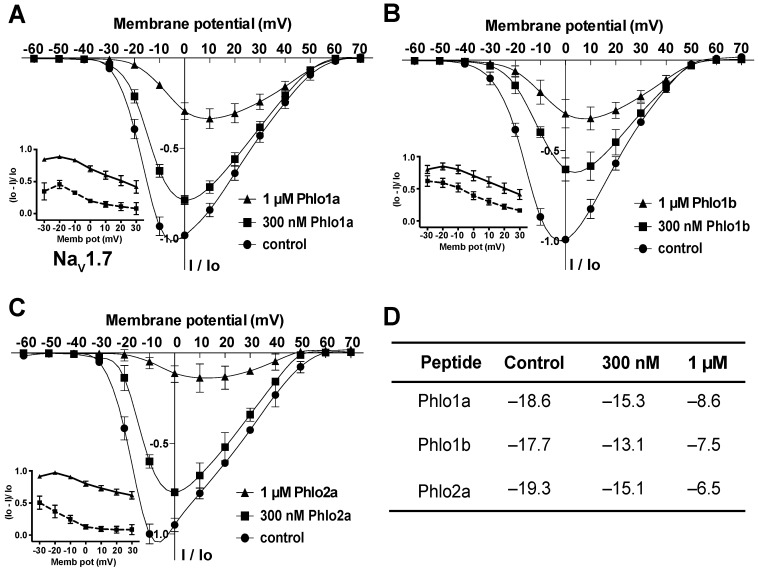
Effect of Phlo1a (**A**), Phlo1b (**B**) and Phlo2a (**C**) on the I-V relationship for hNa_V_1.7. Oocytes were held at −80 mV, and sodium currents were elicited using 50-ms depolarising steps from −60 to +70 mV in 10 mV increments. I-V relationships were obtained in the absence (control, ●) and presence of each peptide at 300 nM (■) and 1 μM (▲). All currents were normalised to the maximum control peak current for each oocyte. Data are mean ± S.E.M. (*n* = 6). Insets in panels A–C show the voltage-dependence of inhibition. (**D**) Quantitation of the effect of each peptide on the V_0.5_ (in mV) of hNa_V_1.7.

Many spider-venom peptides, such as the ceratotoxins (CcoTx1, CcoTx2, CcoTx3), phrixotoxin (PaurTx3), and ProTx-I, inhibit Na_V_ channels by shifting the threshold for channel activation to more positive potentials [26,28,29]. Thus, we investigated the effects of *Phlogius* peptides on the current-voltage (I-V) relationship for hNa_V_1.7 using step-depolarisations ranging from −60 to +70 mV from a holding potential of −80 mV. Figure 10 shows that, under control conditions, the threshold of initial channel activation was approximately −30 mV, the V_0.5_ was about −18 mV, and the peak current was evoked between −10 and −5 mV. All three peptides shifted the V_0.5_ for activation of hNa_V_1.7 to more positive potentials in a concentration-dependent manner; the shift was ~4 mV at 300 nM and 10–12 mV at 1 μM peptide (Figure 10A–C, summarised in Figure 10D). Furthermore, the inhibition of hNa_V_1.7 by all three peptides was voltage-dependent, with lower inhibition at more positive test pulses (insets to Figure 10A–C). Given that Phlo1a, Phlo1b and Phlo2a all cause concentration-dependent, depolarising shifts in the I-V relationship for hNa_V_1.7, we propose that they are gating modifiers that inhibit channel activation via interaction with one or more voltage-sensor domains [15,27,30].

#### 2.3.3. Subtype Selectivity of *Phlogius* Toxins

**Figure 11:**
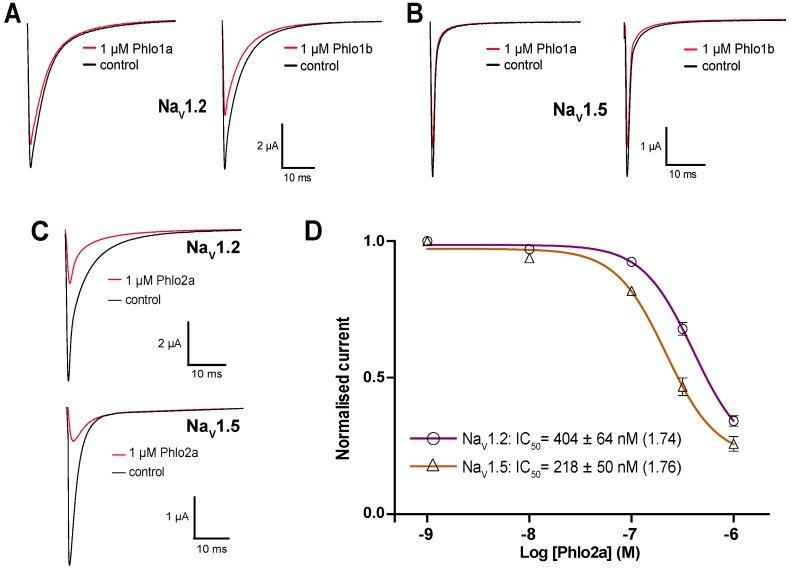
Effects of Phlo1a and Phlo1b on (**A**) rNa_V_1.2 and (**B**) hNa_V_1.5 expressed in *Xenopus* oocytes. Currents were evoked by a 50-ms step depolarisation to 0 mV from a holding potential of −80 mV every 10 s. (**C**) Effect of Phlo2a on rNa_V_1.2 and hNa_V_1.5 currents in *Xenopus* oocytes. (**D**) Concentration-effect curves for inhibition of rNa_V_1.2 and hNa_V_1.5 currents by Phlo2a (*n* = 5). Data are presented as mean ± S.E.M and the Hill coefficients are shown in parentheses.

In order to gain insight into the Na_V_ subtype selectivity of the *Phlogius* peptides, we also examined their effect on rNa_V_1.2 and hNa_V_1.5. Na_V_1.2 is a TTX-sensitive channel that is predominantly expressed in the central nervous system while Na_V_1.5 is a cardiac-specific isoform. Phlo1a inhibited rNa_V_1.2 and hNa_V_1.5 much less potently than hNa_V_1.7 resulting in less than 20% inhibition at 1 μM (Figure 11A,B). At a concentration of 1 μM, Phlo1b had a similar effect as Phlo1a at hNa_V_1.5 but it was slightly more potent at rNa_V_1.2, with 1 μM peptide causing a 37% reduction in currents (Figure 11A,B). This indicates that the single *C*-terminal residue difference between these peptides (Ile to Phe) influences their Nav subtype selectivity. Variations in Na_V_ subtype selectivity due to small sequence variations have been noted previously in spider-venom peptides. Two tarantula toxins isolated from *Ceratogyrus cornuatus* (CcoTx1 and CcoTx2) differ by only one residue, but display dramatic differences in their inhibitory effect on Na_V_1.3 [28]. CcoTx1 does not inhibit Na_V_1.3, whereas CcoTx2 reduces Na_V_1.3 currents with an IC_50_ of 88 nM [28].

In contrast to Phlo1a and Phlo1b, Phlo2a strongly inhibited rNa_V_1.2 and hNa_V_1.5 at a concentration of 1 μM (Figure 11C). The concentration-effect curves obtained for inhibition of rNa_V_1.2 and hNa_V_1.5 by Phlo2a yielded IC_50_ values of 404 ± 64 nM and 218 ± 50 nM, respectively (Figure 11D). These values are very similar to the IC_50_ of 333 nM obtained for inhibition of hNa_V_1.7 by Phlo2a, indicating that this peptide has a low degree of Na_V_ subtype selectivity, which is common for peptides from this toxin family. The most potent blocker of hNa_V_1.7 within this family is β/ω-TRTX-Tp2a (ProTx-II), which inhibits this channel with an IC_50_ of 0.3 nM [31]. However, like Phlo2a, ProTx-II also lacks subtype selectivity and potently inhibits Na_V_1.2 and Na_V_1.5 (IC_50_ = 41 and 79 nM, respectively) [31]. ProTx-II shifts the voltage-dependence of activation of Na_V_1.5 to more positive potentials and has a similar potency to ProTx-I [26]. An extensive mutagenesis study of Na_V_.1.5 led to the conclusion that ProTx-II does not bind to receptor site 4 on the domain II voltage sensor [32], suggesting the existence of a novel toxin-binding site. In contrast, a later study concluded that ProTx-II is gating modifier that reduces sodium conductance by trapping the domain II voltage sensor in the closed state [33]. Consistent with this study, elegant work with chimeric K_V_1.2/Na_V_1.2 chimeric channels indicated that ProTx-II has complex pharmacology and is capable of binding to the voltage sensors in domains I, II (receptor site 4), and IV (receptor site 3) of Na_V_1.2 [27].

Due to the small amounts of native Phlo1a and Phlo1b that were available and their relatively weak activity at rNa_V_1.2 and hNa_V_1.5, we could not obtain complete concentration-effect curves, and consequently the IC_50_ values for these channels remain to be determined. Nevertheless, it is clear that both peptides inhibit hNa_V_1.7 more potently than rNa_V_1.2 and hNa_V_1.5, making them a more promising starting point for development of hNa_V_1.7-selective analgesics than Phlo2a.

We have shown that venom from Australia theraphosid spiders represents an untapped source of potential hNa_V_1.7 inhibitors. Electrophysiology-guided fractionation of venom from a *Phlogius* sp. tarantula led to the isolation of three disulfide-rich peptides that inhibit hNa_V_1.7 with similar IC_50_ values in the range 330–470 nM. All three peptides act as gating modifiers that shift the voltage-dependence of channel activation to more depolarised potentials. As for other members of NaSpTx2 and NaSpTx3, we propose that this occurs by virtue of their binding to one or more of the voltage sensor domains [14,15,27]. One of these peptides (Phlo1a) has a high level of selectivity for Na_V_1.7 over Na_V_1.2 and Na_V_1.5 and thus it represents a good starting point for the rational engineering of subtype-selective inhibitors of Na_V_1.7 for development as analgesics.

Future studies of these peptides will focus on elucidation of structure-function relationships and identification of their binding site on hNa_V_1.7 with a view to rational engineering of more potent and subtype-selective analogues. Overall, the discovery of new Na_V_ channel modulators and the further characterisation of known Na_V_ modulators will extend our understanding of Na_V_ channel function and facilitate the development of new therapeutic treatments.

## 3. Experimental Section

### 3.1. Venom Fractionation and Peptide Purification

Crude venom obtained by electrostimulation was diluted ~100-fold into 10% solvent B (0.043% trifluoroacetic acid (TFA, Auspep, Tullamarine VIC, Australia) in 90% acetonitrile (ACN)), centrifuged (17,000 g, 15 min, 4 °C) and fractioned via RP-HPLC using a Prominence HPLC system (Shimadzu, Kyoto, Japan). Venom (2.5 mg) was loaded onto a to an Agilent C_18_ column (250 × 9.4 mm, 300 Å) and fractionated using the following gradient: 15% solvent B in solvent A (0.05% TFA in water) at a flow rate of 3 mL/min for 2.5 min, followed by a linear gradient of 15%–45% solvent B over 52.5 min, then 45%–70% solvent B over 5 min. Absorbance was monitored at 214 nm and 280 nm using a Shimadzu SPD-10A_VP_ UV-VIS detector. Fractions were collected manually and dried on a vacuum rotary evaporator. The dried fractions were dissolved in water and aliquots of each fraction were assayed for activity against hNa_V_1.7 expressed in *Xenopus* oocytes (see below for details). Active fractions were further separated using a Thermo C_18_ column (150 × 4.6 mm, 5 μm) and a gradient of 20%–50% solvent B over 60 min at a flow rate of 1 mL/min. Peptide purity was verified by RP-HPLC using a Thermo C_18_ column (50 × 2.1 mm, 5μm) with a gradient of 10%–50% solvent B for 16.5 min at a flow rate of 0.25 mL/min. Unless otherwise stated, all reagents were purchased from Sigma, St Louis, MO, USA.

### 3.2. MALDI-TOF Mass Spectrometry

Peptide masses were verified by MALDI-TOF MS using a model 4700 Proteomics Bioanalyser (Applied Biosciences, Foster City, CA, USA). α-Cyano-4-hyroxy-cinnamic acid (CHCA) was used as the matrix. RP-HPLC fractions were mixed 1:1 (*v*/*v*) with CHCA (7.5 mg/mL in 50/50 ACN/H_2_O, 0.1% TFA). MALDI–TOF mass spectra were collected in reflector positive mode and the reported masses are monoisotopic M + H^+^ ions. For sequencing of intact peptides, 1,5-DAN was used as a reductive matrix [34]. The active fractions were mixed 1:1 (*v*/*v*) with 1,5-DAN (15 mg/mL in 50/50 ACN/H_2_O, 0.1% formic acid (FA)).

### 3.3. Reduction/Alkylation of Cysteine Residues

Purified peptides were reduced and alkylated using triethylphosphine and iodoethanol respectively, as previously described [35]. Approximately 3 μg of each pure active peptide was dissolved in 50 μL of 100 mM ammonium carbonate. The reduction/alkylation reagent was prepared by mixing 97.5% ACN, 2% iodoethanol and 0.5% triethylphosphine (*v*/*v*). An equal volume of the reagent was added to the peptide sample and then the reaction mixture was incubated for 2 h at 37 °C. At the end of the incubation period, samples were vacuum-dried on a speedvac for at least 1 h. The dried samples were re-suspended in 0.1% TFA and desalted using RP-HPLC with a Thermo C_18_ column (15%–50% solvent B over 30 min at a flow rate of 1 mL/min). The masses of the reduced/alkylated peptides were determined by MALDI-TOF MS prior to trypsin digestion.

### 3.4. Tryptic Digestion

The reduced and alkylated peptides were digested using a 20:1 (*w*/*w*) ratio of peptide to trypsin (Proteomics Grade, Sigma) in 30 mM ammonium bicarbonate, pH 8. The samples were incubated for 2 h at 37 °C. Digestion was quenched by the addition of 1% FA and the resultant cleavage products were analysed using MALDI-TOF MS. MS ions were selected for tandem mass spectrometry (MS/MS) followed by manual analysis of spectra and comparison to theoretical fragmentation using ProteinProspector Tools [36].

### 3.5. Carboxypeptidase Y Digestion

Reduced and alkylated peptides were dissolved in 15 μL of 100 mM ammonium acetate buffer, pH 5.5. CPY (1 μL of 2 ng/μL; Sequencing Grade, Sigma) was added to the peptide solution. The mixture was left to react at 37 °C and aliquots were taken at 1, 2, 5, 10, 15, 30 and 60 min for MS analysis. The digestion was stopped at the desired time by the addition of 2 μL of 1% FA. MS analysis of the digestion products was performed by mixing 0.5 μL of the digest with 0.5 μL of CHCA matrix. Each dried spot was washed on-plate by adding 0.5 μL of 1% FA, allowing it to permeate the matrix for ~30 s, then excess liquid was removed with a Kimwipe via capillary action.

### 3.6. Preparation and Analysis of Venom-Gland Transcriptome

The venom gland of a single specimen of *Pholius* sp. was dissected four days after depleting the gland of venom by electrostimulation. RNA was extracted using a standard TRIzol (Life Technologies, Carlsbad, CA, USA) protocol, and enriched for poly(A) RNA using an Oligotex mRNA kit (Qiagen, Venlo, Limburg, Netherlands). The resulting mRNA was submitted to the Australian Genome Research Facility (Brisbane, QLD, Australia) where it was reverse transcribed, fragmented, and ligated into a 10-base multiplex identification tag before it was sequenced on a Roche 454 GS FLX+ platform. After removal of low-quality reads, the remaining 72,023 reads were assembled *de novo* using MIRA v3.2.1 (2011) (Open source via http://sourceforge.net/projects/mira-assembler/files/MIRA/Older releases/), resulting in a total of 10,621 contigs (average length 523 bases) and 2904 singlets (average length 335 bases). Open reading frames were predicted, translated and compiled into a local search database using CLC Main Workbench 7 software (CLC bio, Aarhus, Denmark, 2014).

Sequence tags obtained from MALDI-TOF MS analysis were BLAST searched against the *Phlogius* sp. venom-gland transcriptome using CLC Main Workbench 7 software (CLC bio, Aarhus, Denmark, 2014). Peptide sequences were compared with related spider toxins in the ArachnoServer database (www.arachnoserver.org) using the BLAST search form [23]. Multiple sequence alignments were performed using the program ClustalW then manually refined.

### 3.7. Heterologous Expression of Vertebrate Na_V_ Channels in Frog Oocytes

Plasmids containing cloned rNa_V_1.2, hNa_V_1.5 and hNa_V_1.7 were linearised, then capped cRNAs were synthesised using a T7 mMESSAGE-mMACHINE transcription kit (Ambion, Austin, TX, USA). Stage V-VI oocytes were obtained from anesthetised *Xenopus* frogs and prepared as previously described [37]. Oocytes were injected with 20–40 ng of cRNA (Nanoject 2000; WPI, Sarasota, FL, USA) and incubated for 2–6 days at 17 °C in ND96 solution (96 mM NaCl, 2 mM KCl, 1 mM CaCl_2_, 2 mM MgCl_2_, 5 mM HEPES, pH 7.4), supplemented with 2.5 mM sodium pyruvate, 50 μg/mL gentamicin and 2.5% horse serum prior to electrophysiological recordings. All work with animals was carried out in strict accordance with the recommendations in the Australian code of practice for the care and use of animals for scientific purposes. The protocol was approved by the Anatomical Biosciences group of the Animal Ethics Committee at The University of Queensland (Approval Number QBI/059/13/ARC/NHMRC).

### 3.8. Two-Electrode Voltage-Clamp Electrophysiology

Two-electrode voltage clamp (TEVC) recordings were performed at room temperature (20 °C–22 °C) under voltage-clamp (Axoclamp 900A, Molecular Devices, Sunnyvale, CA, USA) using two standard glass microelectrodes of 0.5–1 MΩ resistance when filled with 3 M KCl solution. Stimulation, data acquisition, and analysis were performed using pCLAMP software (Version 10, Molecular Devices, Sunnyvale, CA, USA). Peptide stock solutions were made up to 30 μM, and serial dilutions were prepared in ND96 solution (pH 7.4) containing 0.1% bovine serum albumin (BSA). Venom peptides were applied directly to the recording chamber to prevent adsorption to plastics.

Na_V_ channel recordings were performed on oocytes clamped at −80 mV. Data were sampled at 20 kHz and filtered at 2 kHz. Inward sodium currents were elicited by a 50-ms depolarising step to 0 mV every 10 s. Once the peak current had stabilised (typically ~3 min), serial dilutions of peptides were applied to oocytes to obtain concentration-effect curves. Data were analysed using Clampfit 10.2 and Prism 6.0 (GrahPad Software, La Jolla, CA, USA, 2013). The Hill equation was fit to the data to obtain the half-maximal inhibitory concentration (IC_50_) values and Hill coefficient (*n*H). Data are presented as mean ± S.E.M. (*n* = number of oocytes). I-V relationships in the absence and presence of 300 nM and 1 μM peptide were obtained on oocytes clamped at −80 mV; families of currents were evoked by applying 50-ms depolarising steps from −60 mV to +70 mV with 10-mV increments every 10 s. After acquiring the control I–V curve, the first concentration of peptide was added and the inhibitory effect was allowed to plateau (a test pulse from −80 to 0 mV every 10 s) before obtaining an I–V curve in the presence of peptide. This was repeated for the second concentration of peptide. Data were normalised to the maximal peak current and analysed using Clampfit 10.2 and Prism 6.0.

### 3.9. Deposition of Protein and cDNA Sequence Information

All protein and cDNA sequence information derived for Phlo1a, Phlo1b, and Phlo2a has been submitted to the publicly accessible ArachnoServer spider-toxin database [23,38]. The ArachnoServer accession numbers for Phlo1a, Phlo1b, and Phlo2a are AS002321, AS002322, and AS002323, respectively. Toxin records can be accessed directly using the final four digits of the accession number; for example, for Phlo1a, navigate to www.arachnoserver.org/toxincard.html?id=2321.

## Supplementary Materials

Supplementary materials can be accessed at: http://www.mdpi.com/2072-6651/7/7/2494/s1.
