# Supplementary material for: Three Peptide Modulators of the Human Voltage-Gated Sodium Channel 1.7, an Important Analgesic Target, from the Venom of an Australian Tarantula
Source: Toxins (Basel). 2015 Jun 30;7(7):2494–513. doi: 10.3390/toxins7072494 (PMC4516925; doi:10.3390/toxins7072494)
Supplement: Supplementary file 1 [file toxins-07-02494-s001.pdf]

Supplementary Information

A

```
F18 (Phlo1a): RL9trimmed_s11674_(ORF)
ATGAAGGTTTCAGTACTAATAACTCTTGCTGTATTGGGGGTGATGTTTGTTTGGACTTCTGCTGCT
 m  k  v  s  v  l  i  t  l  a  v  l  g  v  m  f  v  w  t  s  a  A

GAACAAGAAGATCATGGTTCGGATCGAAGGGACTCACCTGCATTGCTAAAAAATCTACTAGGGGAA
 E  Q  E  D  H  G  S  D  R  R  D  S  P  A  L  L  K  N  L  L  G  E

GAAGTATTCCAGTCTGAGGAAAGAGCCTGCAGGGAACTGCTTGGAGGCTGTAGTAAAGATTCCGAT
 E  V  F  Q  S  E  E  R  A  C  R  E  L  L  G  G  C  S  K  D  S  D

TGCTGTGCACACTTGGAATGCCGAAAAAAGTGGCCATATCATTGTGTGTGGGATTGGACCATCGGC
 C  C  A  H  L  E  C  R  K  K  W  P  Y  H  C  V  W  D  W  T  I  G

AAATGA
 K Stp

F19 (Phlo1b): RL9trimmed_rep_79_(ORF)-2
ATGAAGGTTTCAGTACTAATAACTCTTGCTGTATTGGGGGTGATGTTTGTTTGGACTTCTGCTGCT
 m  k  v  s  v  l  i  t  l  a  v  l  g  v  m  f  v  w  t  s  a  A

GAACAAGAAGATCATGGTTCGGATCGAAGGGACTCACCTGCATTGCTAAAATCTCTGGGGAGAGTA
 E  Q  E  D  H  G  S  D  R  R  D  S  P  A  L  L  K  S  L  G  R  V

TTCCAGTCTGAGGAAAGAGCCTGCAGGGAACTGCTTGGAGGCTGTAGTAAAGATTCCGATTGCTGT
 F  Q  S  E  E  R  A  C  R  E  L  L  G  G  C  S  K  D  S  D  C  C

GCACACTTGGAATGCCGAAAAAAGTGGCCATATCATTGTGTGTGGGATTGGACCTTCGGCAATGAG
 A  H  L  E  C  R  K  K  W  P  Y  H  C  V  W  D  W  T  F  G  N  E

AAATCCTGA
 K  S Stp
```

B

```
           1   5    10   15   20   25   30   35   40
Phlo1a: MKVSVLITLAVLGVMFVWTSAAEQEDHGSDRRDSPALLKNLLGEE       45
Phlo1b: MKVSVLITLAVLGVMFVWTSAAEQEDHGSDRRDSPALLKSL-GR-       43

               50   55   60   65   70   75   80   85
Phlo1a: VFQSEERACRELLGGCSKDSDCCAHLECRKKWPYHCVWDWTI*GK      89
Phlo1b: VFQSEERACRELLGGCSKDSDCCAHLECRKKWPYHCVWDWTF*GNEKS   90
```

Figure S1. (**A**) cDNA sequences encoding the complete precursors for Phlo1a and Phlo1b shown above an *in silico* translation of the transcript (bold letters) showing the amino acid sequence of the prepropeptide (bold). The predicted signal sequence is indicated by red lowercase letters, the predicted propeptides are shown in blue uppercase letters, and the mature toxin sequence is shown in green underline uppercase letters. (**B**) Sequence alignment of the Phlo1a and Phlo1b prepropeptide precursors. Colour coding is the same as in panel (**A**). Asterisk indicates C-terminal propeptide cleavage and amidation site.
